# Kallistatin attenuates endothelial senescence by modulating Let‐7g‐mediated miR‐34a‐SIRT1‐eNOS pathway

**DOI:** 10.1111/jcmm.13734

**Published:** 2018-07-11

**Authors:** Youming Guo, Lee Chao, Julie Chao

**Affiliations:** ^1^ Department of Biochemistry and Molecular Biology Medical University of South Carolina Charleston SC USA

**Keywords:** endothelial senescence, eNOS, inflammation, kallistatin, Let‐7g, miR‐34a, oxidative stress, SIRT1

## Abstract

Kallistatin, a plasma protein, protects against vascular and organ injury. This study is aimed to investigate the role and mechanism of kallistatin in endothelial senescence. Kallistatin inhibited H_2_O_2_‐induced senescence in human endothelial cells, as indicated by reduced senescence‐associated‐β‐galactosidase activity, p16^INK^ ^4a^ and plasminogen activator inhibitor‐1 expression, and elevated telomerase activity. Kallistatin blocked H_2_O_2_‐induced superoxide formation, NADPH oxidase levels and VCAM‐1, ICAM‐1, IL‐6 and miR‐34a synthesis. Kallistatin reversed H_2_O_2_‐mediated inhibition of endothelial nitric oxide synthase (eNOS), SIRT1, catalase and superoxide dismutase (SOD)‐2 expression, and kallistatin alone stimulated the synthesis of these antioxidant enzymes. Moreover, kallistatin's anti‐senescence and anti‐oxidant effects were attributed to SIRT1‐mediated eNOS pathway. Kallistatin, via interaction with tyrosine kinase, up‐regulated Let‐7g, whereas Let‐7g inhibitor abolished kallistatin's effects on miR‐34a and SIRT1/eNOS synthesis, leading to inhibition of senescence, oxidative stress and inflammation. Furthermore, lung endothelial cells isolated from endothelium‐specific kallistatin knockout mice displayed marked reduction in mouse kallistatin levels. Kallistatin deficiency in mouse endothelial cells exacerbated senescence, oxidative stress and inflammation compared to wild‐type mouse endothelial cells, and H_2_O_2_ treatment further magnified these effects. Kallistatin deficiency caused marked reduction in Let‐7g, SIRT1, eNOS, catalase and SOD‐1 mRNA levels, and elevated miR‐34a synthesis in mouse endothelial cells. These findings indicate that endogenous kallistatin through novel mechanisms protects against endothelial senescence by modulating Let‐7g‐mediated miR‐34a‐SIRT1‐eNOS pathway.

## 1 INTRODUCTION

Endothelial senescence contributes to the onset and development of vascular injury and age‐associated vascular diseases, such as atherosclerosis, diabetes and coronary artery diseases.^1^, ^2^, ^3^, ^4^ Vascular senescence gives rise to the alteration of morphological features and functional physiology in blood vessels.^5^ The key features of senescent cells are as follows: permanent growth arrest state, flattened morphology, elevation of senescence‐associated‐β‐galactosidase (SA‐β‐gal) activity, cell cycle inhibitor p16^INK4a^ expression and endothelial dysfunction marker plasminogen activator inhibitor‐1 (PAI‐1) expression.^6^, ^7^, ^8^ Moreover, cellular senescence is characterized by shortened telomere length or reduced telomerase activity.^9^ Oxidative stress and inflammation have been implicated in the ageing process and adversely affect endothelial availability and function.^10^, ^11^ Oxidative stress compromises telomere integrity and nitric oxide (NO) bioavailability to accelerate cellular senescence in endothelial cells.^12^ Vascular senescence is also associated with enhanced expression of the inflammatory molecules, vascular cell adhesion molecule‐1 (VCAM‐1) and intercellular adhesion molecule‐1 (ICAM‐1), which leads to augmented adhesion of monocytes into vascular wall, facilitating the ignition and progression of ageing‐associated vascular diseases.^13^, ^14^ Therefore, inhibition of oxidative stress and inflammation is of great significance for controlling endothelial senescence.

MicroRNAs (miRNAs) are essential regulators for a plethora of cellular processes.^15^ A highly evolutionarily conserved miRNA Let‐7g has been reported to inhibit oxLDL‐induced apoptosis of endothelial cells and alleviate atherosclerosis in mice.^16^, ^17^ Let‐7g also has the ability to improve multiple endothelial functions by stimulating sirtuin 1 (SIRT1) signalling.^18^ Furthermore, miR‐34a, a well‐recognized tumour suppressor and a senescent inducer, triggers endothelial senescence by directly suppressing the target gene SIRT1.^19^ SIRT1, an NAD^+^‐dependent deacetylase and anti‐ageing molecule, is decreased in vascular tissue undergoing senescence.^20^ Evidence has indicated a positive feedback regulation between SIRT1 and endothelial nitric oxide synthase (eNOS). SIRT1 promotes the expression of eNOS and activates eNOS by its deacetylase activity, while eNOS through NO production stimulates SIRT1 expression.^21^, ^22^, ^23^ NO produced from eNOS has a wide range of biological properties to maintain vascular homeostasis, including vascular dilation, and inhibition of oxidative stress and inflammation.^24^, ^25^, ^26^ Therefore, Let‐7g, miR‐34a and antioxidant genes SIRT1 and eNOS are key signalling molecules for regulating endothelial senescence.

Kallistatin was discovered in human plasma as a tissue kallikrein‐binding protein and identified as a serine proteinase inhibitor.^27^, ^28^, ^29^, ^30^, ^31^, ^32^ Kallistatin plays a protective role in vascular and organ injury.^33^ Kallistatin gene or protein delivery attenuates vascular and multi‐organ injuries by its pleiotropic activities in vasodilation, and inhibition of oxidative stress, inflammation and apoptosis in animal models and cultured cells.^33^, ^34^, ^35^, ^36^, ^37^, ^38^, ^39^, ^40^ Kallistatin inhibits vascular inflammation and oxidative stress by stimulating eNOS expression and activation, and antagonizing tumour necrosis factor (TNF)‐α‐mediated inflammatory gene expression in endothelial cells.^38^, ^40^, ^41^ Recently, we showed that kallistatin treatment alleviates endothelial progenitor cell (EPC) senescence, mouse aortic aging and prolonged *Caenorhabditis elegans* (*C. elegans)* lifespan by inhibiting miR‐34a and increasing SIRT1 levels.^42^ Moreover, plasma kallistatin levels were shown to be positively associated with leucocyte telomere length in young African Americans, implicating the involvement of kallistatin in anti‐ageing process.^43^ H_2_O_2_ is widely used to achieve oxidative stress‐induced premature senescence in endothelial cells and fibroblasts.^44^, ^45^ According to previous findings, the dose of H_2_O_2_ (100 μmol/L) represents subcytotoxic conditions to induce senescence.^46^, ^47^ In this study, we investigated the role and mechanism of kallistatin in oxidative stress‐induced endothelial senescence by 2 approaches: (1) exogenous kallistatin treatment in human umbilical vein endothelial cells (HUVECs), and (2) endogenous kallistatin deficiency in mouse lung endothelial cells isolated from endothelium‐specific kallistatin‐deficient (KS^endo−/−^) mice.

## 2 MATERIAL AND METHODS

### 2.1 Purification and characterization of recombinant human kallistatin

Recombinant human kallistatin was expressed and secreted into serum‐free medium of cultured HEK293T cells, and purified by ammonium sulphate precipitation followed by nickel‐affinity chromatography as described previously.^30^, ^48^ The purity and identity of human kallistatin were verified by SDS‐polyacrylamide gel electrophoresis and Western blot with a specific monoclonal antibody.^48^, ^49^

### 2.2 Cell culture and treatments

Human umbilical vein endothelial cells (HUVECs) were kindly provided by Dr. Robin Muise‐Helmericks (Medical University of South Carolina). Mouse lung endothelial cells were isolated from wild‐type (WT) mice and endothelial‐specific kallistatin deficient mice (KS^endo−/−^ mice). HUVECs and mouse lung endothelial cells were cultured in endothelial basal medium (EBM)‐2 with 10% foetal bovine serum (FBS) and supplements (Lonza, Alpharetta, GA, USA), and maintained in a humidified atmosphere of 5% CO_2_ in air at 37°C. HUVECs at 80% confluency were pretreated with kallistatin (1 μmol/L) followed by hydrogen peroxide (H_2_O_2,_ 100 μmol/L; Fisher Scientific) challenge for 3 days in serum‐free EBM‐2 medium; senescent markers and oxidative stress indicators were evaluated. To measure gene expression, HUVECs were preincubated with kallistatin (1 μmol/L) followed by H_2_O_2_ (100 μmol/L) treated for 24 hours. To determine the causal relationship of SIRT1 and eNOS, HUVECs were pretreated with SIRT1 inhibitor nicotinamide (NAM, 5 mmol/L; Sigma, St. Louis, MO, USA) or NOS inhibitor Nω**‐**nitro‐L‐arginine methyl ester hydrochloride (L‐NAME, 100 μmol/L; Sigma) for 30 minutes, followed by kallistatin (1 μmol/L) and H_2_O_2_ (100 μmol/L) treatment for 24 hours. Similarly, cultured primary mouse lung endothelial cells at 80% confluency were treated with or without H_2_O_2_ (100 μmol/L) for 3 days to evaluate senescent markers and oxidative stress indicators, and for 24 hours to measure gene expression.

### 2.3 Senescence‐associated β‐galactosidase staining

Cellular senescence of HUVECs or mouse lung endothelial cells in 12‐well plates was determined with senescence‐associated β‐galactosidase (SA‐β‐gal) staining kit (Cell Signaling, Danvers, MA, USA). The number of positive SA‐β‐gal cells was observed by light microscopy in 10 randomly chosen fields and expressed as a percentage of counted cells.

### 2.4 Telomerase activity assay

Human umbilical vein endothelial cells were preincubated with or without 1 μmol/L kallistatin for 30 minutes prior to the addition of H_2_O_2_ (100 μmol/L) for 24 hours. Cells were lysed with non‐denaturing lysis buffer. Telomerase activity was measured using TRAPEZE RT telomerase detection kit (EMD Millipore, Billerica, MA, USA).

### 2.5 NADPH oxidase activity assay

The enzymatic activity of NADPH oxidase was assessed by a luminescence assay in the presence of lucigenin (250 μmol/L; Sigma) and NADPH (100 μmol/L; Sigma) as described.^50^ Fluorescence intensity was continuously monitored for 15 minutes with a TD20/20 luminometer. Protein concentrations were measured by Pierce BCA protein assay kit (Thermo Fisher Scientific, Bremen, Germany). The chemiluminescent signal was normalized by protein concentration.

### 2.6 Detection of superoxide formation

Cellular superoxide generation was detected using the fluorescent probe 2′,7′‐dichlorodihydrofluorescein diacetate (DCFH‐DA; Sigma) as described.^38^ Treated HUVECs in six‐well plates were incubated for 30 minutes with DCFH‐DA (10 μmol/L). Fluorescence was examined and imaged by fluorescence microscopy. To quantify levels of reactive oxygen species (ROS), HUVECs were seeded onto a 96‐well fluorescence plate and treated as above. Relative fluorescence was measured using the fluorescence plate reader (Biotek, Winooski, VT, USA) at 485 nm excitation and 535 nm emission. Superoxide levels in mouse lung endothelial cells were determined by fluorescent probe dihydroethidium (DHE; Sigma). Briefly, treated mouse lung endothelial cells in six‐well plates were incubated with DHE (3 μmol/L) in a light protected humidified chamber at 37°C for 30 minutes. Ethidium fluorescence was examined and imaged by fluorescence microscopy, and comparative levels of fluorescence were analysed using ImageJ software [National Institutes of Health (NIH), Bethesda, MD, USA].

### 2.7 miRNA transfection

Human umbilical vein endothelial cells were grown in antibiotic‐free EBM‐2 on plates for 24 hours, and then transfected with Let‐7g inhibitor (10 pmol/L, anti‐sense RNA Let‐7g), miR‐34a mimic or control miRNA (10 pmol/L; Life Technologies, Grand Island, NY, USA) with Lipofectamine RNAiMAX reagent (Life Technologies) according to the manufacturer's protocol. After 12 hours of transfection, cells were washed and cultured in serum‐free EBM‐2 medium with or without kallistatin (1 μmol/L) or H_2_O_2_ (100 μmol/L). After 24 hours, cells were washed and processed for RNA or protein analysis.

### 2.8 Quantitation of mRNA by real‐time polymerase chain reaction (qRT‐PCR)

Total RNA was extracted using Trizol according to manufacturer's instruction. Total RNA was reverse‐transcribed using the High‐Capacity cDNA Reverse Transcription Kit (Applied Biosystems, Foster City, CA, USA) or miRNA Reverse Transcription Kit (Applied Biosystems). Real‐time PCR was performed with Taqman Gene Expression Assay kit (Applied Biosystems). Human 18S and U6 were used as internal control genes for quantifying mRNA and miRNA expression in HUVECs, respectively. GAPDH and U6 were used as internal controls for quantifying mouse mRNA and miRNA expression. The following primers were used: 18S (Hs99999901_s1), p16^INK4a^ (Hs00923894_m1), eNOS (Hs01574659_m1), SIRT1 (Hs01009006_m1), catalase (Hs00156308_m1), PAI‐1 (Hs01126606_m1), superoxide dismutase (SOD)‐2 (Hs00167309_m1), ICAM‐1 (Hs00277001_m1), interleukin (IL)‐6 (Hs00174131_m1), VCAM‐1 (Hs00365486_m1), NADPH oxidase 4 (Hs00418356_m1), miR‐34a (002316), Let‐7g (002282), mouse GAPDH (Mm99999915_g1), mouse eNOS (Mm00435217_m1), mouse SIRT1 (Mm00490758_m1), mouse catalase (Mm00437992_m1), mouse SOD‐1 (Mm01344233_g1), mouse VCAM1 (Mm01320970_m1), mouse IL‐6 (Mm00446190_m1), mouse ICAM‐1 (Mm00516024_g1), mouse miR‐34a (465771), mouse kallistatin (Mm00434669_m1), mouse PAI‐1 (Mm00435858_m1), mouse p16^INK4a^ (Mm00494449_m1). Data were analysed with 2^−ΔΔCt^ value calculation using control genes for normalization.

### 2.9 Western blot

Cells were lysed in lysis buffer supplemented with 1 mmol/L PMSF, 1 mmol/L Na_3_VO_4_, 1 mmol/L NaF and a protease inhibitor cocktail (Thermo Fisher Scientific). Proteins from cell lysates were separated by 10% SDS‐polyacrylamide gel electrophoresis and transferred to a PVDF membrane (Bio‐Rad Laboratories, Hercules, CA, USA). After being blocked in 7% nonfat milk, protein blots were probed with a primary antibody overnight followed by incubation with a horseradish peroxidase‐conjugated secondary antibody (Santa Cruz Biotechnology, Dallas, TX, USA). Primary antibodies were rabbit anti‐SIRT1, mouse monoclonal anti‐eNOS, rabbit antimouse kallistatin, mouse monoclonal anti‐β‐actin and rabbit anti‐α‐tubulin. Chemiluminescence was detected by the ECL‐plus kit (GE Healthcare, Waukesha, WI, USA), and band densitometry was carried out by ChemiDoc MP imaging system (Bio‐Rad Laboratories).

### 2.10 Generation of endothelium‐specific kallistatin knockout mice

Heterozygous kallistatin floxing mice in C57/BL6 strain (male and female, KS^fl/+^) were obtained from Biocytogen, LLC (Worcester, MA, USA), and male wild‐type C57/BL6 mice (7‐8 weeks of age) were purchased from Harlan (Indianapolis, IN, USA). These mice were housed in a germ‐free environment. All procedures complied with the standard for care and use of animal subjects as stated in the *Guide for the Care and Use of Laboratory Animals* (Institute of Laboratory Resources, National Academy of Sciences, Bethesda, MD, USA). KS^fl/+^ mice were inbred to produce homozygous floxed kallistatin mice (KS^fl/fl^). To generate endothelium‐specific heterozygous kallistatin knockout mice (Tie2Cre^+^KS^fl/+^), female KS^fl/fl^ mice were crossed with male Tie2‐Cre mice (The Jackson Laboratories, Bar Harbor, ME, USA). Tie2Cre^+^KS^fl/+^ mice were then crossed with KS^fl/fl^ mice to generate endothelium‐specific kallistatin knockout (KS^endo−/−^) mice. The expression of mouse kallistatin null allele in endothelium of KS^endo−/−^ mice was determined by PCR with a pair of custom primers: 5′‐TCAAGTCCCTCATGTGCGTTGGTAG‐3′ and 5′‐ATGTCAATCACAATGCCCTCTGCCT‐3′. The amplification conditions are 95°C for 5 minutes and 35 cycles of 95°C for 30 seconds, 63°C for 30 seconds and 72°C for 1 minute, and a 10‐minute incubation at 72°C at the end of the run. Amplification products were resolved on a 3% agarose gel. Mouse kallistatin deficiency in isolated mouse lung endothelial cells was verified by qRT‐PCR and Western blot.

### 2.11 Isolation and Identification of mouse lung endothelial cells

Mouse lung endothelial cells were isolated from 8‐week‐old KS^endo−/−^ and WT mice as described.^51^ Mice were euthanized by CO_2_ inhalation followed by cervical dislocation. The lungs were excised, chopped and digested with 0.1% collagenase in phosphate‐buffered saline for 45 minutes. The lung‐digestive solution was quenched with complete‐DMEM and filtered through a 70‐μm tissue sieve followed by restrain with a 40‐μm cell strainer for multiple passages. The cell suspension was isolated by magnetic immunoselection with CD31‐conjugated magnetic beads (Miltenyi Biotec, San Diego, CA, USA) using the magnetic‐activated cell sorting system (MACS, Miltenyi Biotec). Purified mouse lung endothelial cells were cultured on gelatin‐coated tissue culture dishes with EBM‐2 medium with 10% FBS and 1% antibiotics until they became confluent. Newly grown colonies were digested, incubated with CD31 magnetic beads and selected through MACS again. After the second selection, mouse lung endothelial cells were seeded in a T75 flask provided with complete EBM‐2 medium to grow robustly. Mouse endothelial cells were characterized with endothelial marker CD31 by PCR and immunostaining. For immunostaining, mouse endothelial cells were fixed with 4% paraformaldehyde, incubated with blocking buffer and followed with rat antimouse CD31 antibody (Santa Cruz Biotechnology) overnight at 4°C. After washing with phosphate‐buffered saline (PBS), cells were incubated with anti‐rat FITC secondary antibody (Sigma) for 1 hour at room temperature, washed with PBS and counterstained with Hoechst 33342 (5 μg/mL; Sigma). Cells were photographed by a fluorescence microscope.

### 2.12 Statistical analysis

Data are presented as mean ± SEM. Statistical analyses were performed with GraphPad Prism 7 (GraphPad Software, La Jolla, CA, USA). ANOVA and Student's *t* test analysis were used to assess differences between data means as appropriate. A value of *P* < .05 was considered statistically significant.

## 3 RESULTS

### 3.1 Kallistatin attenuates H_2_O_2_‐induced senescence in human endothelial cells

**Figure 1:**
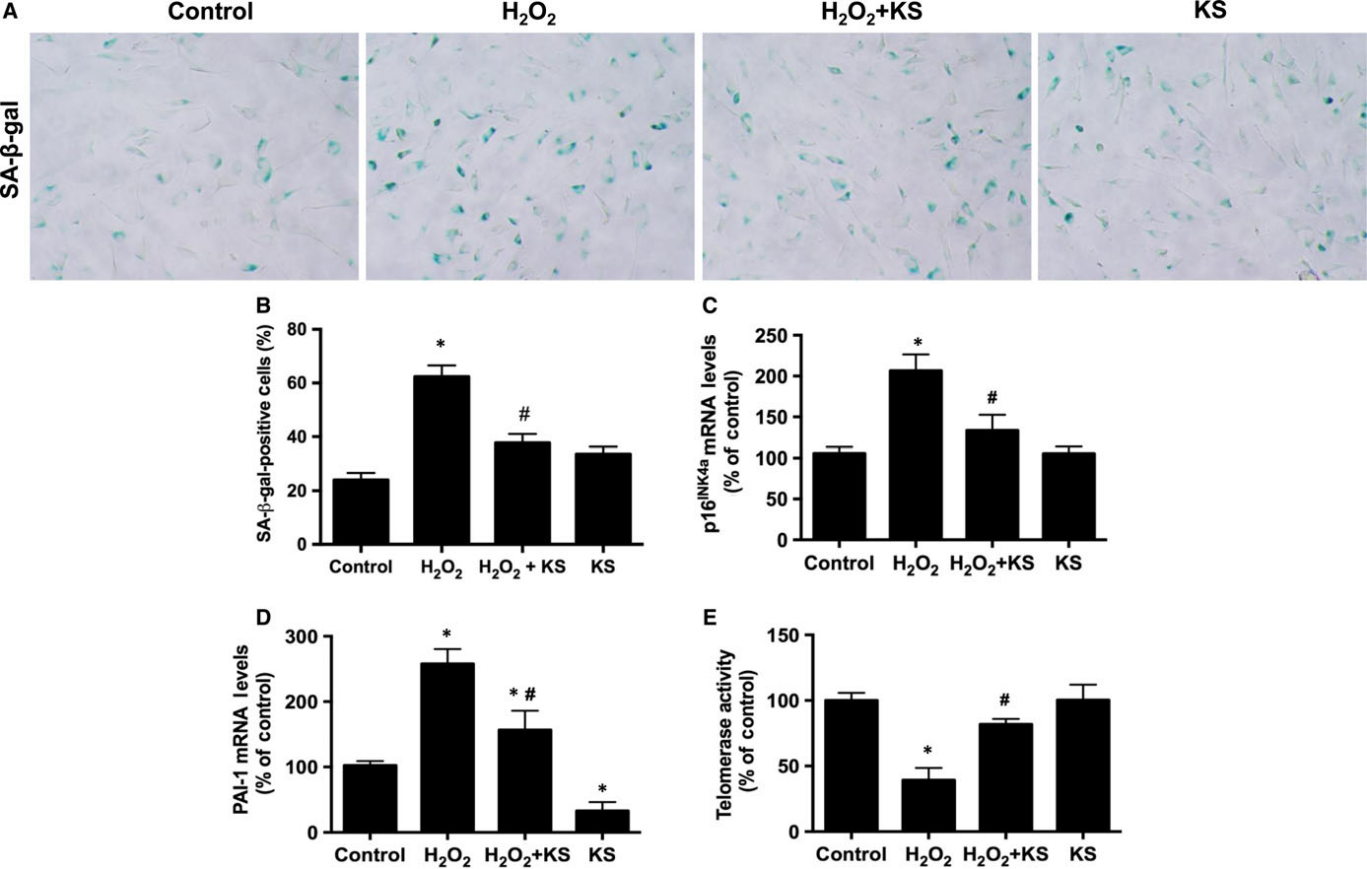
Kallistatin (KS) alleviates H_2_O_2_‐induced endothelial senescence in human endothelial cells. Representative images of SA‐β‐gal staining (A), and quantitative analysis of positive SA‐β‐gal staining cells (B). p16^INK^ ^4a^ and PAI‐1 mRNA levels analysed by qRT‐PCR (C, D). Telomerase activity (E). Values are expressed as mean ± SEM. (n = 3 in each group). *P < .05 vs control, ^#^ *P* < .05 vs H_2_O_2_ group

Representative images showed that H_2_O_2_ treatment induced pronounced cellular senescence in HUVECs, characterized by increased senescent marker SA‐β‐gal activity compared with the control group, while purified recombinant human kallistatin pretreatment reversed the effect (Figure 1A). Quantitative analysis of positive SA‐β‐gal staining cells confirmed the results (Figure 1B). Senescent markers p16^INK4A^, PAI‐1 and telomerase were also evaluated. Kallistatin blocked H_2_O_2_‐induced p16^INK4A^ and PAI‐1 expression, and prevented H_2_O_2_‐mediated inhibition of telomerase activity (Figure 1C‐E). Therefore, kallistatin is capable of blocking oxidative stress‐induced endothelial senescence.

### 3.2 Kallistatin inhibits H_2_O_2_‐induced oxidative stress and inflammation

**Figure 2:**
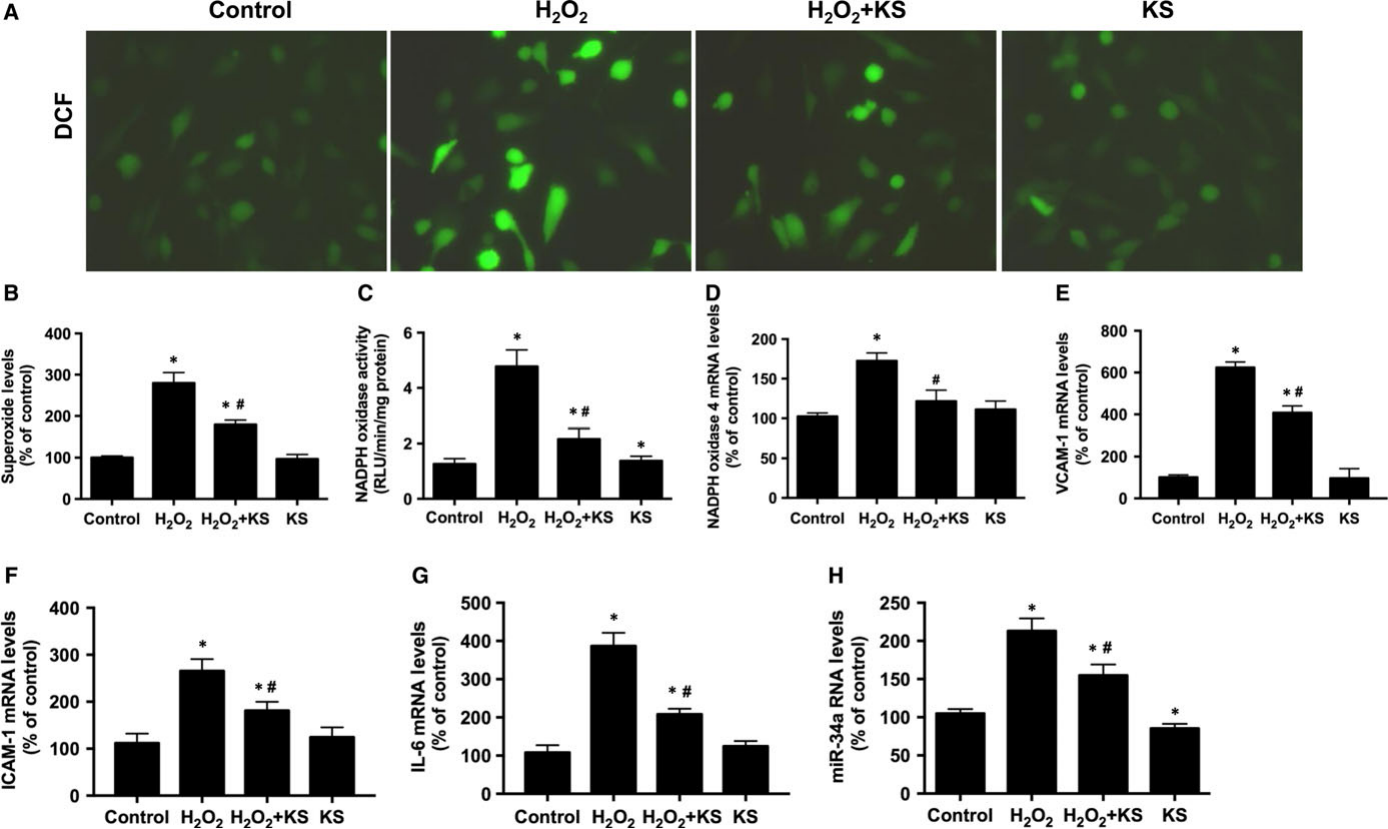
Kallistatin (KS) suppresses H_2_O_2_‐induced oxidative stress and inflammation in human endothelial cells. Representative images of superoxide formation determined by fluorescence dye DCF (A), and quantitative analysis of DCF fluorescence (B). NADPH oxidase activity (C). mRNA levels of NADPH oxidase 4 (D), VCAM‐1 (E), ICAM‐1 (F), IL‐6 (G) and miR‐34a (H) analysed by qRT‐PCR. Values are expressed as mean ± SEM. (n = 3 in each group). *P < .05 vs control, ^#^ *P* < .05 vs H_2_O_2_ group

Next we assessed the effect of kallistatin on H_2_O_2_‐induced oxidative stress and inflammation. Representative images showed that kallistatin attenuated H_2_O_2_‐induced superoxide formation in human endothelial cells, which was verified by quantitative analysis (Figure 2A,B). Similarly, kallistatin antagonized H_2_O_2_‐induced NADPH oxidase activity and NADPH oxidase 4 mRNA levels (Figure 2C,D). Moreover, kallistatin inhibited H_2_O_2_‐mediated expression of the pro‐inflammatory genes VCAM‐1, ICAM‐1 and IL‐6 (Figure 2E‐G). Kallistatin alone inhibited miR‐34a and prevented H_2_O_2_‐induced miR‐34a synthesis (Figure 2H). These results indicate that kallistatin inhibits H_2_O_2_‐induced oxidative stress and inflammation in endothelial cells.

### 3.3 Kallistatin inhibits endothelial senescence and oxidative stress through SIRT1‐mediated eNOS pathway

**Figure 3:**
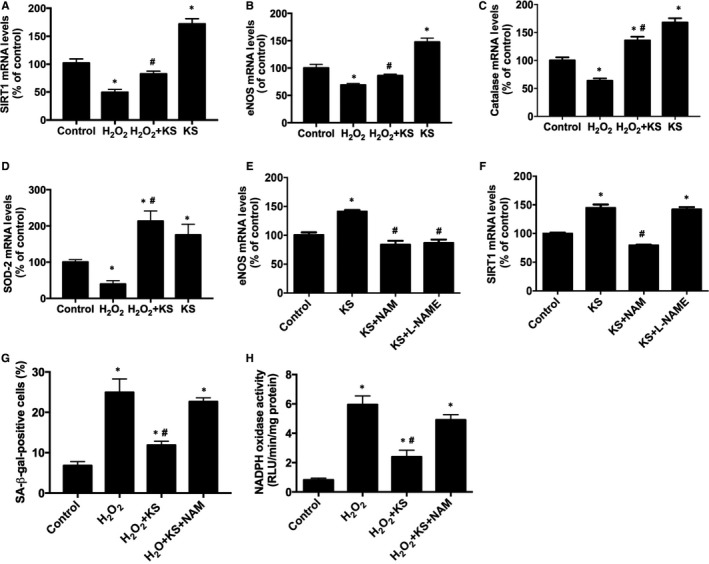
Kallistatin (KS) inhibits H_2_O_2_‐induced senescence and oxidative stress through SIRT1‐eNOS pathway in human endothelial cells. The effect of KS on SIRT1, eNOS, catalase and SOD‐2 mRNA levels analysed by qRT‐PCR (A‐D). The effects of SIRT1 inhibitor NAM and NOS inhibitor L‐NAME on KS‐mediated eNOS and SIRT1 expression (E, F). The effect of NAM on KS‐regulated SA‐β‐gal activity (G) and NADPH oxidase activity (H). Values are expressed as mean ± SEM. (n = 3 in each group). *P < .05 vs control, ^#^ *P* < .05 vs KS group or H_2_O_2_ group

To determine whether the kallistatin‐mediated protection against endothelial senescence is associated with antioxidant gene regulation, the effects of kallistatin on the regulation of SIRT1, eNOS, catalase and SOD‐2 were determined. Kallistatin treatment not only markedly enhanced SIRT1, eNOS, catalase and SOD‐2 expression, but also prevented H_2_O_2_‐mediated inhibition of these antioxidant genes (Figure 3A‐D). Moreover, NAM, a potent inhibitor of SIRT1 activity, blocked kallistatin‐stimulated eNOS expression, whereas L‐NAME, an NOS inhibitor, had no effect on kallistatin‐stimulated SIRT1 expression (Figure 3E,F), indicating that kallistatin via SIRT1 elevation stimulates eNOS. Furthermore, NAM abolished kallistatin's antioxidant activity and anti‐senescent action, as evidenced by reversing kallistatin's inhibition of SA‐β‐gal activity and NADPH oxidase activity in HUVECs (Figure 3G,H). These results indicate that the SIRT1‐mediated eNOS pathway plays a crucial role in kallistatin's inhibition of endothelial senescence and oxidative stress.

### 3.4 Kallistatin by Let‐7g up‐regulation blocks miR‐34a‐mediated inhibition of SIRT1‐eNOS pathway, and reduces endothelial senescence, oxidative stress and inflammation

**Figure 4:**
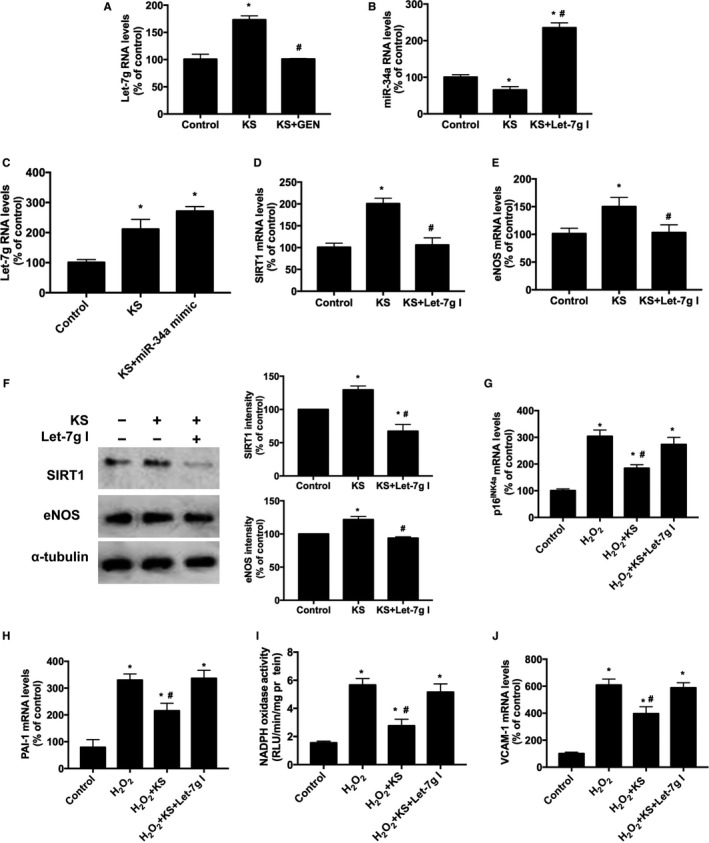
Kallistatin (KS) stimulates Let‐7g synthesis to up‐regulate SIRT1 and eNOS and inhibit endothelial senescence, oxidative stress and inflammation. The effect of tyrosine kinase inhibitor genistein (GEN) on KS‐regulated Let‐7g synthesis was determined by qRT‐PCR (A). The effect of Let‐7g inhibitor (Let‐7g I) on KS‐regulated miR‐34a synthesis (B), and the effect of miR‐34a mimic on KS‐induced Let‐7g synthesis (C) determined by qRT‐PCR. The effect of Let‐7g inhibitor on KS‐regulated SIRT1 (D) and eNOS mRNA levels (E) analysed by qRT‐PCR; and SIRT1 and eNOS protein levels by Western blot and quantitative analysis (F). The effects of Let‐7g inhibitor on KS‐regulated mRNA levels of senescent markers p16^INK^ ^4a^ and PAI‐1 (G, H); NADPH oxidase activity (I) and inflammatory gene VCAM‐1 mRNA levels (J). Values are expressed as mean ± SEM. (n = 3/group). *P < .05 vs control, ^#^ *P* < .05 vs KS group or H_2_O_2_ group

To illustrate whether kallistatin‐mediated inhibition of endothelial senescence was associated with up‐regulation of Let‐7g, the effect of kallistatin on Let‐7g expression was determined. Kallistatin treatment significantly increased Let‐7g synthesis, whereas genistein, a tyrosine kinase inhibitor, blocked kallistatin's effect (Figure 4A), implicating that kallistatin interacts with a cell surface tyrosine kinase to stimulate Let‐7g synthesis. Let‐7g inhibitor (Let‐7g anti‐sense RNA) blocked kallistatin's inhibition of miR‐34a synthesis (Figure 4B), indicating that kallistatin regulates Let‐7g‐mediated miR‐34a pathway. Conversely, miR‐34a mimic had no effect on kallistatin‐induced Let‐7g expression (Figure 4C); thus, kallistatin's effect on Let‐7g up‐regulation is independent of miR‐34a. Moreover, Let‐7g inhibitor abolished kallistatin‐induced SIRT1 and eNOS mRNA levels, and blocked kallistatin‐mediated elevation of SIRT1 and eNOS levels by western blot (Figure 4D‐F), indicating an essential role of Let‐7g in kallistatin's induction of antioxidant genes. Furthermore, Let‐7g inhibitor abolished kallistatin's inhibitory effect on H_2_O_2_‐induced senescence, characterized by elevated p16^INK4a^ and PAI‐1 mRNA levels in endothelial cells (Figure 4G,H). Likewise, Let‐7g inhibitor blocked kallistatin's effect on H_2_O_2_‐induced NADPH oxidase activity and VCAM‐1 expression (Figure 4I,J). These combined findings indicate that kallistatin, by interacting with a tyrosine kinase, up‐regulates Let‐7g, thus inhibiting miR‐34a‐SIRT1‐eNOS pathway. Therefore, Let‐7g plays a crucial role in mediating kallistatin's inhibitory effects on endothelial senescence.

### 3.5 Identification of kallistatin deficiency in mouse lung endothelial cells

**Figure 5:**
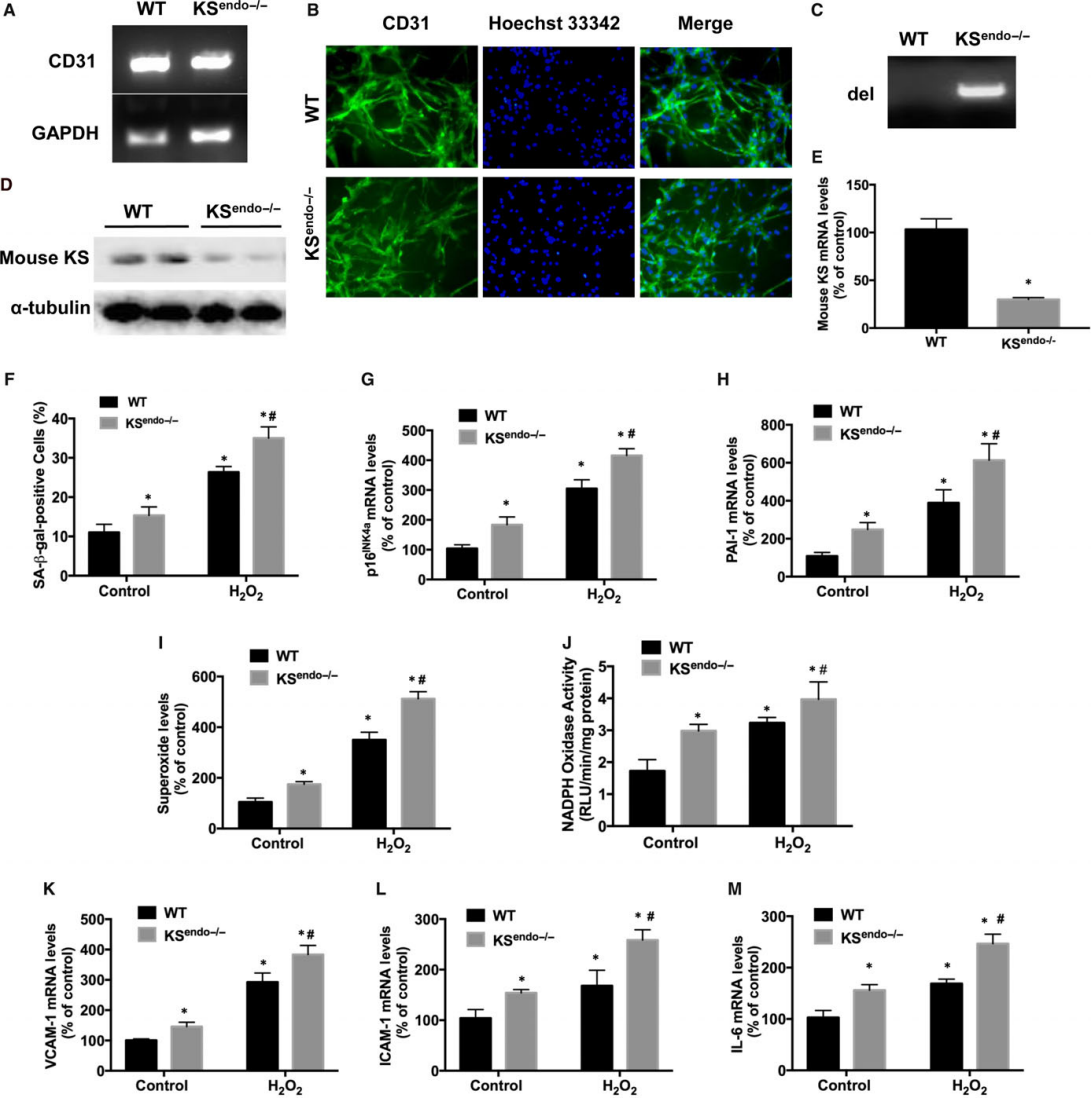
Kallistatin (KS) deficiency in mouse lung endothelial cells increases senescence, oxidative stress and inflammation. Endothelial marker CD31 expression in mouse endothelial cells determined by PCR (A) (n = 3/group). Representative images of immunostaining of CD31 (green), with nuclei (Hoechst 33342, blue) and their merged images are shown (B) (n = 3/group). Identification of mouse kallistatin depletion (del) by target null gene DNA electrophoresis (C) (n = 3/group). Representative immunoblots of mouse KS protein (D) (n = 3/group), and mouse KS mRNA levels (E) (n = 6/group). Quantitative analysis of SA‐β‐gal activity (F), p16^INK^ ^4a^ and PAI‐1 mRNA levels (G, H), superoxide formation (I), NADPH oxidase activity (J),VCAM‐1, ICAM‐1 and IL‐6 mRNA levels (K‐M) in mouse endothelial cells with or without H_2_O_2_ treatment (n = 6/group). Values are expressed as mean ± SEM. *P < .05 vs WT control group, ^#^ *P* < .05 vs WT H_2_O_2_ group

We generated endothelium‐specific kallistatin knockout (KS^endo−/−^) mice by crossing KS^fl/fl^ mice with Tie2Cre^+^KS^fl/+^ mice. Lung endothelial cells were isolated from WT mice and KS^endo−/−^ mice. Cultured primary mouse lung endothelial cells were identified by CD31 expression and immunostaining (Figure 5A,B). In lysed mouse endothelial cell DNA samples, kallistatin null allele (del) was detected only in KS^endo−/−^ mice, but not in WT mice (Figure 5C), indicating that endogenous kallistatin is ablated in mouse endothelial cells. Moreover, the protein and mRNA levels of mouse kallistatin were markedly diminished in endothelial cells from KS^endo−/−^ mice, compared with endothelial cells from WT mice (Figure 5D,E). These results confirm that mouse endogenous kallistatin is deficient in KS^endo−/−^ mouse endothelial cells.

### 3.6 Kallistatin deficiency aggravates oxidative stress‐induced senescence, oxidative stress and inflammation in mouse endothelial cells

Cultured primary mouse lung endothelial cells with or without H_2_O_2_ treatment were used to determine the effect of kallistatin deficiency on endothelial senescence, oxidative stress and inflammation. In the non‐H_2_O_2_‐treated groups, kallistatin‐deficient endothelial cells displayed increased SA‐β‐gal activity, p16^INK4a^ and PAI‐1 expression, compared to WT mouse endothelial cells (Figure 5F‐H). Mouse kallistatin deficiency in H_2_O_2_‐treated endothelial cells further enhanced the levels of senescence makers, compared to H_2_O_2_‐treated WT mouse endothelial cells (Figure 5F‐H). Moreover, cellular superoxide formation and NADPH oxidase activity were increased upon H_2_O_2_ treatment, and kallistatin deficiency further enhanced superoxide formation and NADPH oxidase activity (Figure 5I,J). Likewise, kallistatin deficiency increased VCAM‐1, ICAM‐1 and IL‐6 mRNA levels in kallistatin‐deficient endothelial cells treatment compared with WT endothelial cells with or without H_2_O_2_ (Figure 5K‐M). Therefore, kallistatin deficiency in mouse endothelial cells exacerbates endothelial senescence, oxidative stress and inflammation. These findings implicate a protective role of endogenous kallistatin in stress‐induced endothelial senescence.

### 3.7 Kallistatin deficiency reduces Let‐7g and antioxidant gene expression and increases miR‐34a synthesis in mouse endothelial cells

**Figure 6:**
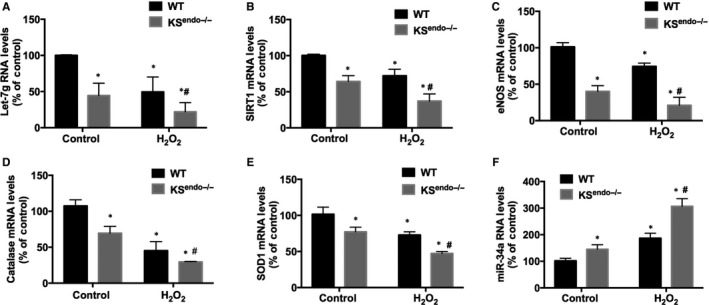
Kallistatin (KS) deficiency inhibits the synthesis of Let‐7g and antioxidant genes and elevates miR‐34a synthesis in mouse lung endothelial cells. PCR analysis of Let‐7g (A), SIRT1 (B), eNOS (C), catalase (D), SOD‐1 (E) and miR‐34a (F) in WT and KS ^endo−/−^ mouse lung endothelial cells treated with or without H_2_O_2_. Values are expressed as mean ± SEM. (n = 3/group). *P < .05 vs WT control group, ^#^ *P* < .05 vs WT H_2_O_2_ group

Kallistatin deficiency in KS^endo−/−^ mouse endothelial cells caused a significant reduction in Let‐7g, SIRT1, eNOS, catalase and SOD‐1 expression compared with WT mouse cells (Figure 6A‐E). Moreover, H_2_O_2_ treatment reduced the expression of Let‐7g and antioxidant genes in endothelial cells from WT mice, and kallistatin deficiency further reduced the expression of these genes (Figure 6A‐E). Conversely, kallistatin deficiency increased miR‐34a synthesis in mouse lung endothelial cells, compared with WT endothelial cells, with or without H_2_O_2_ treatment (Figure 6F). These results indicate that endothelial senescence in kallistatin‐deficient mouse endothelial cells is attributed to down‐regulation of Let‐7g and antioxidant genes, and elevation of miR‐34a levels.

## 4 DISCUSSION

**Figure 7:**
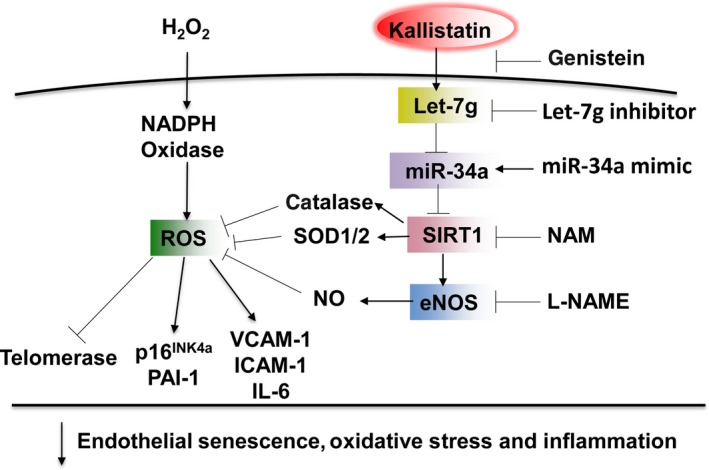
Signalling pathway by which kallistatin inhibits endothelial senescence, oxidative stress and inflammation. Kallistatin through tyrosine kinase stimulates Let‐7g synthesis, inhibits miR‐34 and up‐regulates SIRT1‐mediated‐eNOS pathway, thereby elevating catalase, SOD‐1/2 and NO levels, leading to blockade of H_2_O_2_‐modulated ROS formation, p16^INK^ ^4a^, PAI‐1, VCAM‐1, ICAM‐1 and IL‐6 expression, and telomerase activity in endothelial cells. Genistein, tyrosine kinase inhibitor; NAM, SIRT1 inhibitor; L‐NAME, NOS inhibitor

This study demonstrates that endogenous kallistatin plays a protective role in endothelial senescence, and kallistatin through a novel mechanism inhibits cellular senescence, oxidative stress and inflammation. Kallistatin antagonized oxidative stress‐induced senescence, as indicated by inhibiting p16^INK4a^ and PAI‐1 levels and increasing telomerase activity in human endothelial cells. Moreover, kallistatin, via tyrosine kinase, up‐regulated Let‐7g, thus preventing miR‐34a‐mediated inhibition of the SIRT1‐eNOS pathway. Activation of SIRT1‐eNOS signalling resulted in increased catalase, SOD‐1/2 and NO levels, and decreased ROS formation, VCAM‐1, ICAM‐1 and IL‐6 expression. The signalling mechanism by which kallistatin inhibits endothelial senescence, oxidative stress and inflammation is shown in Figure 7. To confirm the pivotal role of endogenous kallistatin in protection against endothelial senescence, we generated endothelial‐specific kallistatin knockout (KS^endo−/−)^ mice and isolated lung endothelial cells. The protective actions of endogenous kallistatin in vascular injury and senescence were validated by exacerbated senescence, oxidative stress and inflammation in mouse endothelial cells from KS^endo−/−^ mice. As endothelial senescence is a key contributor to vascular dysfunction and the ageing process, the current study indicates an important role of kallistatin in vascular ageing and age‐related vascular diseases.

Oxidative stress and inflammation are hallmarks of endothelial senescence.^52^, ^53^ Kallistatin administration has been shown to attenuate vascular injury and organ damage and ageing, in conjunction with reduced oxidative stress and inflammation in animal models with hypertension, arthritis, diabetes and sepsis.^37^, ^38^, ^39^, ^40^, ^48^, ^54^ In this study, we showed that kallistatin inhibited H_2_O_2_‐induced oxidative stress and pro‐inflammatory gene expression, including VCAM‐1, ICAM‐1 and IL‐6, in human endothelial cells. Moreover, kallistatin stimulated antioxidant gene expression, including SIRT1, eNOS, catalase and SOD‐2. NO production by eNOS inhibits superoxide formation and inflammation.^25^, ^26^ Catalase, one of SIRT1's downstream antioxidant enzymes, is decreased in H_2_O_2_‐induced premature senescence.^55^ Human SOD‐2 and mouse SOD‐1 are antioxidant enzymes and play an important role in the prevention of endothelial senescence and inflammation.^56^, ^57^ Previous reports indicated a reciprocal positive feedback regulation between SIRT1 and eNOS.^21^, ^22^, ^23^ SIRT1 has been shown to induce eNOS expression and activity, and eNOS through NO formation up‐regulates SIRT1 and maintains vascular homoeostasis.^21^, ^22^, ^23^, ^24^ Kallistatin was shown to increase SIRT1 and eNOS synthesis in human endothelial cells and EPCs.^42^, ^58^ Moreover, kallistatin treatment increased eNOS activity and NO formation in human endothelial cells.^38^ The present study shows that kallistatin stimulates SIRT1‐mediated eNOS pathway, as kallistatin‐induced eNOS expression was blocked by a SIRT1 inhibitor, but kallistatin‐mediated SIRT1 expression was not affected by a NOS inhibitor. Collectively, kallistatin through stimulating SIRT1‐mediated eNOS pathway protects against senescence, oxidative stress and inflammation.

Many senescence‐associated miRNA signatures have been identified and reported.^59^, ^60^ The present study mainly focused on studying the effect of kallistatin on differential regulation of 2 miRNAs, Let‐7g and miR‐34a in endothelial senescence. Let‐7g was recently recognized as an anti‐aging miRNA to improve endothelial functions.^18^ Let‐7g negatively regulated apoptosis, senescence and inflammation in endothelial cells.^16^, ^17^, ^18^ Up‐regulation of SIRT1 levels was involved in Let‐7g ‐mediated inhibition of endothelial senescence.^18^ In this study, we showed that kallistatin increased Let‐7g synthesis, and kallistatin, via Let‐7g induction, stimulated SIRT1 and eNOS, leading to inhibition of endothelial senescence, oxidative stress and inflammation in endothelial cells. Importantly, kallistatin harbours 2 structural elements, an active site and a heparin‐binding domain.^30^, ^31^, ^32^ Kallistatin's active site is essential for eNOS and SIRT1 expression through interacting with a tyrosine kinase in EPCs and endothelial cells.^42^, ^58^ Kallistatin via its active site stimulates tyrosine kinase‐PKC‐ERK signalling, leading to the synthesis of suppressor of cytokine signaling‐3 (SOCS3), an anti‐inflammatory gene in cultured macrophages.^61^ It is likely that kallistatin via the active site interacts with tyrosine kinase to stimulate Let‐7g synthesis. Moreover, miR‐34a‐SIRT1 axis has been regarded to be one of critical pathways to regulate endothelial senescence.^19^ Our previous study showed that kallistatin reduced EPC senescence and aorta ageing by inhibiting the synthesis of miR‐34a, a pro‐senescent miRNA.^42^ Consistently, the current study indicates that kallistatin treatment inhibited miR‐34a and prevented H_2_O_2_‐induced miR‐34a synthesis in endothelial cells. Importantly, we found that Let‐7g is the upstream regulator of miR‐34a in response to kallistatin treatment. These results indicate that kallistatin protects against oxidative stress‐induced endothelial senescence by up‐regulating Let‐7g to inhibit miR‐34a‐SIRT1‐eNOS pathway.

To further investigate the role of endogenous kallistatin in vascular senescence, we generated KS^endo−/−^ mice by loxp/Cre technology, and isolated lung endothelial cells from KS^endo−/−^ mice and WT mice. Kallistatin deficiency in mouse endothelial cells accelerated replicative senescence and superoxide formation. Upon H_2_O_2_ treatment, kallistatin deficiency further aggravated stress‐induced senescence, oxidative stress and inflammation, indicating a protective role of endogenous kallistatin in maintaining endothelial viability and function. Furthermore, exacerbated senescence in KS^endo−/−^ endothelial cells was associated with elevation of miR‐34a, and reduction in Let‐7g, SIRT1, eNOS, catalase and SOD‐1. Thus, Let‐7g‐mediated SIRT1‐eNOS pathway is suppressed when kallistatin is deficient. We previously showed that human kallistatin protein treatment reduced vascular senescence and ROS formation, but increased SIRT1 and eNOS levels in aortas of diabetic mice.^42^ Moreover, kallistatin gene delivery decreased aortic superoxide levels and glomerular endothelial loss in hypertensive deoxycorticosterone acetate‐salt rats.^38^ Conversely, kallistatin depletion by neutralizing kallistatin antibody injection exacerbated renal and cardiovascular oxidative stress, inflammation and organ injury in hypertensive rats.^50^ These combined findings support the notion that endogenous kallistatin acts as a protective molecule in vascular injury and senescence by stimulating Let‐7g, SIRT1 and eNOS, and suppressing miR‐34a synthesis.

Overall, this study indicates that kallistatin inhibits cellular senescence, oxidative stress and inflammation by promoting Let‐7g‐mediated inhibition of miR‐34a‐SIRT1‐eNOS pathway in human endothelial cells. Moreover, endogenous kallistatin plays a protective role in endothelial senescence, oxidative stress and inflammation, as confirmed by kallistatin‐deficient mouse endothelial cells. Therefore, kallistatin could potentially be used as a promising new therapeutic strategy for vascular ageing and dysfunction in humans.

## CONFLICT OF INTEREST

None.
